# Manipulating the Phytic Acid Content of Rice Grain Toward Improving Micronutrient Bioavailability

**DOI:** 10.1186/s12284-018-0200-y

**Published:** 2018-01-11

**Authors:** Ishara Perera, Saman Seneweera, Naoki Hirotsu

**Affiliations:** 1 Graduate School of Life Sciences, Toyo University, 1-1-1 Izumino, Itakura-machi, Oura-gun, Gunma, 374-0193 Japan; 2 Centre for Crop Health, University of Southern Queensland, Toowoomba, QLD 4350 Australia; 3 Faculty of Life Sciences, Toyo University, 1-1-1 Izumino, Itakura-machi, Oura-gun, Gunma, 374-0193 Japan

**Keywords:** Bioavailability, Biosynthesis, Gene, Phytic acid, Rice

## Abstract

*Myo-*inositol hexaphosphate, also known as phytic acid (PA), is the most abundant storage form of phosphorus in seeds. PA acts as a strong chelator of metal cations to form phytate and is considered an anti-nutrient as it reduces the bioavailability of important micronutrients. Although the major nutrient source for more than one-half of the global population, rice is a poor source of essential micronutrients. Therefore, biofortification and reducing the PA content of rice have arisen as new strategies for increasing micronutrient bioavailability in rice. Furthermore, global climate change effects, particularly rising atmospheric carbon dioxide concentration, are expected to increase the PA content and reduce the concentrations of most of the essential micronutrients in rice grain. Several genes involved in PA biosynthesis have been identified and characterized in rice. Proper understanding of the genes related to PA accumulation during seed development and creating the means to suppress the expression of these genes should provide a foundation for manipulating the PA content in rice grain. Low-PA rice mutants have been developed that have a significantly lower grain PA content, but these mutants also had reduced yields and poor agronomic performance, traits that challenge their effective use in breeding programs. Nevertheless, transgenic technology has been effective in developing low-PA rice without hampering plant growth or seed development. Moreover, manipulating the micronutrient distribution in rice grain, enhancing micronutrient levels and reducing the PA content in endosperm are possible strategies for increasing mineral bioavailability. Therefore, a holistic breeding approach is essential for developing successful low-PA rice lines. In this review, we focus on the key determinants for PA concentration in rice grain and discuss the possible molecular methods and approaches for manipulating the PA content to increase micronutrient bioavailability.

## Background

*Myo*-inositol 1,2,3,4,5,6-hexa*kis*phosphate (InsP6), commonly known as phytic acid (PA) is the principle storage form of phosphorus (P) in cereal grains, and may account for 65%–85% of the total seed P (Raboy 2000). The remaining P is in the form of soluble inorganic phosphate (Pi: approximately 5%) and cellular P (approximately 10 to 20% of the total seed P) that is found in nucleic acids, proteins, lipids and sugars (Larson et al. 2000). PA is negatively charged and, thus, strongly chelates cations such as calcium (Ca), magnesium (Mg), potassium (K), iron (Fe) and zinc (Zn) and usually exists as mixed salts referred to as phytate or phytin (Raboy 2003). These very insoluble salts prevent the absorption of important nutrients in the human intestine (Mitchikpe et al. 2008) that may then lead to micronutrient deficiencies. The daily intake of PA is found to vary with the age (Amirabdollahian and Ash 2010), country (Ma et al. 2007) and the physiological stage of an individual (Al Hasan et al. 2016; Niknamian and Niknamian 2016). The average PA intake of people in developing countries is higher than that of developed countries due to differences in dietary patterns; i.e., vegetarian diets predominant in the developing countries leading to high levels of PA ingestion (Kwun and Kwon 2000; Amirabdollahian and Ash 2010).

Micronutrient deficiency or hidden hunger, is a global health problem caused by inadequate intake of essential vitamins and minerals from the diet. One in three people of all ages in the world is affected by this nutritional challenge with Fe and Zn deficiencies being the most serious (WHO 2002); however, micronutrient deficiency is more widespread in developing countries where plant-based diets are widely consumed (IFPRI 2016). Among the cereals, rice (*Oryza sativa* L*.*) is one of the most important staple foods for nearly one-half of the global population and the most important crop in Asia (FAO 2013). Although rice is the major source of energy, protein and minerals for mankind, the grain does not provide sufficient amounts of essential micronutrients to fulfill the daily human nutritional requirements compared to other cereals, especially for rice eating populations (Juliano 1993). Hence, enriching rice with essential micronutrients, i.e., Fe and Zn biofortification, is identified as a major strategy to overcome micronutrient malnutrition especially in developing countries (Bouis and Saltzman 2017).

The phosphorus and inositol in PA are generally not bioavailable to non-ruminant animals who lack the digestive enzyme phytase that hydrolyzes P from the PA molecule (Mroz et al. 1994; Marounek et al. 2010). In addition, PA inhibits enzymes needed for protein degradation in the stomach and small intestine (Kies et al. 2006). Ruminants are readily able to digest PA because rumen microorganisms produce phytase. To overcome P deficiency in non-ruminants, phytase is usually given as a supplement (Pontoppidan et al. 2007), which then results in excess P excretion leading to environmental problems, such as eutrophication. Therefore, a low-PA (*lpa*) rice bran would be of greater value for non-ruminant livestock feeds, including poultry, swine, and fish feeds, than would brans derived from normal rice. Furthermore, the rising carbon dioxide concentration ([CO_2_]) in the atmosphere and predicted global warming are expected to influence global crop production. Elevated atmospheric [CO_2_] will also affect grain micronutrient concentration and will increase the PA content (Myers et al. 2014; Dietterich et al. 2015). In spite of these dire predictions, the need for developing cereals and grains with low-PA levels has not been given the attention this important matter requires.

Effective approaches for improving P-utilization and reducing the environmental impact while reducing the effect of PA are envisioned by developing low-PA crops by plant breeding, improving fertilizer management and by optimizing food processing techniques (Erdal et al. 2002; Bentsink et al. 2003; Bregitzer and Raboy 2006; Liang et al. 2008). However, the progress in developing *lpa* rice is modest compared to the success made for maize or barley (Ockenden et al. 2004; Kim et al. 2008), and efforts to improve micronutrient bioavailability in rice have seemed a challenge until the present (Larson et al. 2000; Liu et al. 2007). Over the past decade, several physiological, genetic and molecular studies have been carried out to biofortify rice grains with Zn and Fe. Although there are many published studies on Fe and Zn uptake, translocation inside the plant, grain loading and biofortification of rice grains (Cakmak et al. 2010; Murgia et al. 2012; Nakandalage et al. 2016), information on lowering the PA content of rice with improved bioavailability is very limited. A better understanding of the molecular and physiological basis of PA biosynthesis, distribution of grain PA, effects of genetic and environmental factors on PA accumulation and possible ways to increase micronutrient bioavailability by lowering the effects of PA is essential for developing low-PA crops. This review focuses on physiological, genetic and molecular aspects of PA and the key factors affecting the PA concentration of rice grain. We also discuss strategies for developing low-PA rice to increase micronutrient bioavailability.

## Review

### Antinutrient Effects of PA

The major concern of having PA in the diet is its negative effect on the bioavailability of several minerals. PA is enzymatically hydrolyzed by phytase to lower inositol phosphates, inositol pentaphosphate (InsP5), inositol tetraphosphate, inositol triphosphate, inositol diphosphate and inositol monophosphate, during grain storage, fermentation, germination, food processing and digestion in the human gut (Burbano et al. 1995; Azeke et al. 2011; Hayakawa et al. 2014). However, only InsP6 and InsP5 have a major inhibitory effect on mineral bioavailability (Sandberg et al. 1999). Micronutrient malnutrition affects over three billion people in the world (WHO 2002); most notably, Zn and Fe deficiencies are reported to be linked to high PA intake (Al Hasan et al. 2016). The relatively low bioavailability of these essential minerals is a global nutritional issue at present. For example, Fe is an essential element in hemoglobin, myoglobin and the cytochromes and is important for cognitive development and metabolic functions, whereas Fe deficiency is recognized as the most prevalent nutritional problem in the world (WHO 2015), causing approximately 0.8 million deaths annually (WHO 2002). PA binds to Fe ions in the grain, thereby inhibiting Fe absorption (Iwai et al. 2012). Zinc is a co-factor of more than 300 enzymes important in the physiologies of both plants and animals (McCall et al. 2000) and is an essential micronutrient in cereal grains. Zinc is required for proper function of the immune system and for growth of human tissues. Zinc deficiency causes growth retardation, immune dysfunction, increased mortality, and adverse effects on neurological system development (Wuehler et al. 2007). PA is a key determining factor for Zn absorption (Couzy et al. 1998) and is also reported to react with some proteins and disturb proteolysis through altering protein structure (Kies et al. 2006). Negative effects of PA, especially low bioavailability of minerals, has a significantly greater effect on infants, pregnant and lactating women when cereal-based food is a large portion of their diet (Chan et al. 2007; Al Hasan et al. 2016). Therefore, groups most vulnerable to micronutrient deficiencies, young children, pregnant and lactating women, should consume a low-PA diet to facilitate higher mineral absorption. Moreover, reducing PA also seems important for increasing the bioavailability of available minerals in cereal grains for both human and animal nutrition.

In spite of the negative effects on human nutrition, PA is also reported to be beneficial as a natural plant antioxidant and as a protector against oxidative stress in seeds (Doria et al. 2009). In yeast, PA is involved in mRNA export and translation, RNA editing, DNA repair and vesicular trafficking (Saiardi et al. 2002; Bolger et al. 2008). The negative impacts of PA in nutrition have been emphasized, whereas the beneficial role of PA on plant biology has been underplayed. More attention should also be focused on retaining the beneficial effects of PA in concert with efforts to modify the concentration or the distribution pattern of PA in grain.

### PA in Rice

PA is a naturally occurring compound in plant seeds, mainly in cereals, legumes, oilseeds and nuts and is a common constituent of plant-derived food (Lolas et al. 1976; Garcia-Estepa et al. 1999). In contrast to other small-grain cereals such as wheat, P accumulates in rice plants throughout all stages of development (Rose et al. 2010). P is primarily concentrated in the leaves at the tillering stage, moves to and accumulates in the stems during node elongation until the booting stage, and finally translocate to the panicles where it localizes mostly in the seeds at maturity (Delin and Zhaomin 1996). Phosphorus in the grain is the result of both P uptake at the post-flowering stage (exogenous accumulation) and P remobilization from vegetative parts of the plant (endogenous accumulation) (Julia et al. 2016). Studies with dry bean genotypes suggested that if sufficient P is absorbed during the early growth stages, P can be easily redistributed to growing organs that translocate P to seeds where the nutrient is immediately converted to PA (Coelho et al. 2002). This observation indicates that there is a correlation between the PA content and the P content in seeds. Additionally, the P content in different plant organs is also important in determining the grain PA content. During germination, phytate is degraded by the action of phytases, providing P, mineral cations and *myo*-inositol to the growing seedlings (Afify et al. 2011). However, the mechanism for P loading to the grain and the relative contributions of endogenous and exogenous processes for producing grain PA are not completely understood.

### Determinants of PA Content

The PA content of seeds is primarily influenced by genetic and environmental factors (Liu 2005). Environmental factors broadly include climatic conditions, crop and fertilizer management practices and soil characteristics including soil physical, chemical and biological factors (Dintzis et al. 1992; Kaya et al. 2009; Dhole and Reddy 2015; Brankovic et al. 2015). Also, anticipated climate change effects, especially rising temperature and [CO_2_], may affect the PA content in seeds (Seneweera and Norton 2011; Fernando et al. 2014). However, information about the effect of genetic and environmental factors on P uptake, translocation, remobilization, P partitioning in the rice grain and PA accumulation in rice grain is limited.

#### Effect of Soil Environment (Fertilizer, Physicochemical and Biological Factors)

Phosphorus fertilizer is the second most widely used fertilizer after nitrogen (FAO 2016) and is an essential macronutrient required for vegetative and reproductive plant growth (Hajabbasi and Schumacher 1994). Continuous rice cultivation without supplemental P fertilizer causes depletion of soil P levels (Srilatha and Sharma 2015). Furthermore, as most of the P is stored in the grain, harvesting the crop leads to continuous removal of P taken up by the plant. Consequently, P fertilizer application is required to address soil P deficiencies that could have adverse consequences on crop growth.

Rice plants usually absorb P from both the soil and P fertilizer, but if the soil-available P content is high, plants will absorb more P from the soil and decrease their uptake of P fertilizer; thus, the contribution from P fertilizer to rice yield is reduced (Delin and Zhaomin 1996). Moreover, P availability in wetland rice soils is much higher than in upland soils because reducing conditions facilitate P solubility and thus improve P uptake by plants growing in flooded conditions (Hajiboland et al. 2009). As a result, grain P concentration tends to be higher under lowland conditions than under upland conditions, irrespective of whether P fertilizer has been applied (Somaweera et al. 2015). Interestingly, the soil P fraction is believed to be positively correlated with the PA content in rice grain (Rose et al. 2016). Moreover, the P concentration of different plant parts varies with the P concentration in the soil (Seneweera and Conroy 1997). Furthermore, arbuscular mycorrhizal fungi also contribute significantly to the uptake of P in lowland rice (Hajiboland et al. 2009). In addition, decreases in root-zone oxygen are reported to negatively affect P uptake (Insalud et al. 2006). Studies with pearl millet have shown that application of P increased the concentrations of PA in the grain between 25 and 29%, increased the PA:Zn molar ratio and decreased Zn concentrations between 6 and 11% due to greater P uptake and a dilution of Zn by the large yield increases after P application (Buerkert et al. 1998). On the other hand, intensive P fertilizer application raises more environmental issues, especially when P is leached into water sources. Soil P or P fertilizer seem to be important for controlling grain PA since approximately 70% of the total grain P is stored as PA; however, the contribution of available P or P-fertilizer in P loading to rice grain and practical recommendations for P fertilizer applications that would reduce grain PA without decreasing plant growth and grain yield still remain to be identified.

#### Effect of Elevated [CO_2_] on PA Content

Global [CO_2_] is expected to reach 550 ppm by year 2050 from the present level of 400 ppm, increasing at the rate of 1.5 to 1.8 μmol mol^−1^ yr.^−1^ (IPCC 2013). Rising [CO_2_] is likely to increase plant yield and affect the physical and chemical properties of rice grain (Seneweera 2011; Seneweera and Norton 2011; Myers et al. 2014). The yield enhancements caused by elevated [CO_2_] are mainly due to promoting photosynthetic rates and possibly reducing crop water use (Hasegawa et al. 2013). In contrast, a decrease in the concentrations of most nutrients such as nitrogen (N), P, K, Ca, sulfur (S), Mg, Fe and Zn were observed in most cereals including rice grown in elevated [CO_2_] conditions (Myers et al. 2014). It has been reported that P demand is increased at elevated [CO_2_] which is partly due to stimulation in photosynthesis and plant growth (Seneweera et al. 1996; Kumar et al. 2012). At elevated [CO_2_], a 1.2% increase in phytate content in rice grains was observed compared to that from plants grown in ambient [CO_2_] (Myers et al. 2014). This finding may be explained by enhanced grain yield and suggested the existence of a critical requirement for increased P at elevated [CO_2_] (Seneweera et al. 1994) that must be acquired from the available P pool in the soil (Coelho et al. 2002). These findings suggest that plants will require a large amount of P at elevated [CO_2_] and later can remobilize P into the grain with part of the P content present as PA-P. Phosphorus partitioning to different rice plant organs varies at elevated [CO_2_] with reduced P concentrations in leaf sheaths, leaf blades and roots, although the total uptake remains unaffected (Seneweera and Conroy 1997). In contrast, the micronutrient concentrations, mainly Fe and Zn, decrease at elevated [CO_2_] in the most important staple crops, including rice (Dietterich et al. 2015). A decrease in Zn content of 3.3% (*p* < 0.001) and a decrease in Fe content of 5.2% (p < 0.001) were observed in rice grown at elevated [CO_2_] (Myers et al. 2014). The mechanism(s) responsible for the decline in micronutrient concentration at elevated [CO_2_] was hypothesized to be associated with CO_2_-stimulated carbohydrate production (Myers et al. 2014). However, if a nutrient was diluted by elevated [CO_2_], all other nutrients should change by a similar magnitude; however, this was not the case in most studies (Seneweera 2011; Fernando et al. 2014). Furthermore, alterations in root morphology, quantity of root exudates and increases in rooting depth may be observed in an elevated [CO_2_] environment (Rogers et al. 1992; Nie et al. 2013). The results of a free-air CO_2_ enrichment (FACE) study revealed a 37% increase in total root dry mass in spring wheat during the stem elongation stage (Wechsung et al. 1999). Increases in root length (+10%) and root dry weight (+43%) were observed in soybeans grown in a [CO_2_] of 700 μmol mol^−1^ (Rogers et al. 1992). The exudates from roots of shortleaf pine (*Pinus echinatu* Mill.) increased after 34 weeks of growth under elevated [CO_2_] (Norby et al. 1987). Changes in root morphological and functional traits may allow plants to acquire additional P to meet the increased demand. However, despite the consensus that P acquisition must increase due to growth and yield enhancements, there is no information on the role of P uptake and transporter genes performing at elevated [CO_2_] conditions. This limitation emphasizes the necessity for advanced physiological, chemical and molecular analyses to demonstrate how elevated [CO_2_] affects the use and acquisition of P by plants.

#### Genotypic Variation of PA in Rice

**Fig. 1:**
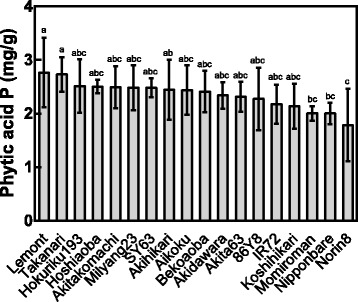
PA-P content of different rice cultivars. The raw data were taken from experiments conducted in Tsukuba, Japan (Dietterich et al. 2015). The average value of the PA-P content and standard deviation for each cultivar are presented in the figure. Means followed by the same letter denote no significant differences according to Tukey’s test (*P* < 0.05)

Breeding programs have been primarily concentrated on increasing the grain yield potential; attention directed toward improving grain quality traits, especially lowering grain PA, has not been a priority during the past few decades. However, recent research has focused on decreasing the grain PA level while maintaining the potential for high grain yield. Therefore, as a primary step, it is important to study different genotypes in diverse climatic conditions and evaluate their genetic variability and environmental interactions related to PA and Pi content and yield traits to identify better lines for breeding. In a study that evaluated brown rice grains from three *indica* rice cultivars, ZN 7, ZN 60 and ZN 34, PA contents of 7.34 mg/g, 3.99 mg/g and 6.79 mg/g were reported, respectively (Wang et al. 2011). Among the 24 *japonica* rice cultivars studied by Liu (2005), the PA content ranged from 0.68% for ‘Xiu 217’ to 1.03% for ‘Huai 9746’. Spikelet architecture changes panicle morphology that can cause grain PA variation in rice depending on the cultivar (Su et al. 2014). The variation in PA-P content among 18 different rice cultivars grown in Tsukuba, Japan was evident in an experiment conducted using FACE technology (Fig. 1). These studies indicated that considerable genotypic variation exists for this trait in rice. Whether this variation is heritable and, if so, the proportion of genetic variability responsible for expression of the trait remain unknown at present.

#### Genetic Factors other than PA-related Genes that Influence the PA Content of Rice Grain

Genotype significantly affects P uptake, concentration and distribution in rice grain (Ren et al. 2006; Tian et al. 2016). Root characteristics, including root morphology and the length and density of root hairs, affect the acquisition of P ions from the soil solution (Hammond et al. 2009; Vejchasarn et al. 2016). Phosphorus is taken up in the inorganic form by roots mainly by diffusion rather than mass flow (Oliveira et al. 2010) and uptake of these ions is accomplished through active absorption via Pi transporters belonging to the phosphate transporter (PT) family (Jia et al. 2011; Liu et al. 2011). To date, 13 plasma membrane-mediated Pi transporters belonging to the PHT1 family (OsPT1-OsPT13) have been identified in rice (Liu et al. 2011). OsPT1 is constitutively expressed in roots and is important for Pi acquisition regardless of Pi availability (Sun et al. 2012), whereas OsPT2 and OsPT6 function in Pi uptake and translocation throughout the plant (Ai et al. 2009). OsPT4 is a functional Pi transporter localized mainly in the plasma membrane of rice root exodermis cells; overexpression of the *OsPT4* gene resulted in higher Pi concentrations in brown rice (Ye et al. 2015). Moreover, OsPT8, OsPT9 and OsPT10 are high-affinity Pi transporters important for Pi uptake in rice (Jia et al. 2011; Wang et al. 2014). Among the reported rice PTs, OsPT11 was shown to play a major role in the arbuscular mycorrhizal Pi uptake pathway (Paszkowski et al. 2002; Yang et al. 2012). The absorbed P enters the epidermal and cortical cells of roots (Santner et al. 2012), is transported to shoots and to the above-ground organs, including seeds, and is synthesized into PA. A SULTR-like phosphorus distribution transporter (SPDT), which encodes a plasma membrane-localized transporter for P, controls the allocation of P to the grains (Yamaji et al. 2017). Knockout mutants of SPDT alter the distribution of P, resulting in a 20% reduction in total P and an approximately 30% reduction in the grain PA concentration.

#### PA-related Genes in Rice

**Fig. 2:**
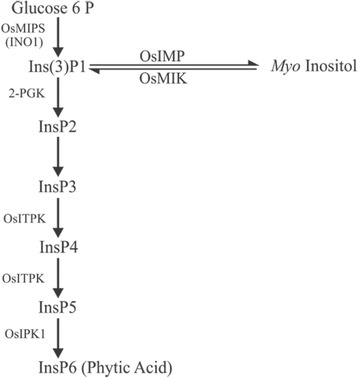
Schematic diagram of the lipid-independent PA biosynthetic pathway in rice seeds. Genes involved in the steps of PA biosynthesis are illustrated

**Fig. 3:**
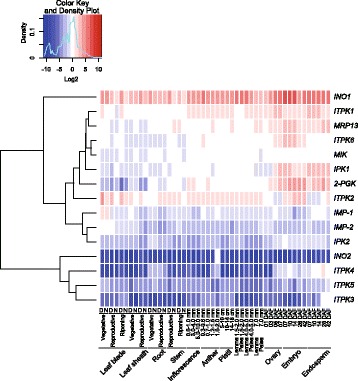
Heat map of PA biosynthetic and transporter genes and their expression profiles among various organs at different developmental stages of the rice plant. A total of 15 PA-biosynthetic and transporter genes identified from the Rice Microarray database (RiceXpro) were analyzed by hierarchical clustering. A heat map was created using the spatio-temporal gene expression values of various organs throughout plant development in the field (Sato et al. 2013) with the heatmap.2 function from the gplot package in R (version 3.2.1). High expression values are shown in red. D; day, N; night, DAF; days after flowering

PA biosynthesis initiates at 4 days after flowering (DAF) in rice (Yoshida et al. 1999) and continues in the aleurone layer and the embryo throughout seed development until 25 DAF (Iwai et al. 2012). In plants, there are two pathways to PA synthesis; namely a lipid-dependent pathway that is common in plant tissues and a lipid-independent pathway that predominates in the seeds of cereals and legumes (Shi et al. 2005; Suzuki et al. 2007; Bhati et al. 2014). Several genes that may be involved in the metabolism of inositol phosphates and PA accumulation have been identified in rice including, *2-phosphoglycerate kinase (2-PGK), inositol monophosphatase (IMP), inositol-pentakisphosphate 2-kinase 1 (IPK1), 1D–myo-inositol 3-phosphate synthase (MIPS), inositol polyphosphate 2-kinase 2 (IPK2), inositol 1,3,4-triskisphosphate 5/6-kinase (ITP5/6 K) and myo-inositol kinase (MIK)* (Suzuki et al. 2007; Kim et al. 2008a, b). The first step of inositol biosynthesis in developing seed is conversion of glucose 6-phosphate (G6P) to *myo*-inositol 3-phosphate (Ins(3)P1) by *MIPS* (Mitsuhashi et al. 2008), followed by a series of phosphorylation steps (Fig. 2). Of the two *Ins(3)P1 synthase* genes reported, *INO1(RINO1)* located on chromosome 3, plays an important role in PA biosynthesis and is expressed in developing seed embryos and in the aleurone layer of rice (Yoshida et al. 1999; Kuwano et al. 2006). The biosynthetic pathway and the enzymes involved in the process of PA synthesis from Ins(3)P1 seem to be complicated and are not fully understood (Kuwano et al. 2009). OsIPK1 catalyzes the last step, resulting in the production of InsP6 from Ins (1,3,4,5,6) P5 (Suzuki et al. 2007). Consistently low levels of PA in the inner endosperm during seed development as measured by ion chromatography suggested that PA synthesized in the inner endosperm may be transported immediately to the aleurone layer and/or PA synthesis occurs in the outer endosperm (Iwai et al. 2012). During seed development, the *IPK2* genes are involved in the lipid-dependent PA biosynthetic pathway in seeds (Suzuki et al. 2007). Nevertheless, the PA biosynthetic pathway in developing seeds is not completely characterized (Shi et al. 2005). In the Rice Annotation Project Database, RAP-DB (Sakai et al. 2013), we found at least 15 genes putatively involved in PA biosynthesis and transport (Table 1). The rice microarray database, RiceXPro (Sato et al. 2013) reveals that these genes have different expression patterns among organs; some genes are expressed in developing seeds in the ovary, embryo and endosperm (Fig. 3).

**Table 1:** List of the Genes Responsible for PA Biosynthesis and Transport in Rice

Gene name	Gene Symbol	RAP-ID	Position	MSU ID
*Myo-inositol 3-phosphate synthase-1*	*INO1*	Os03g0192700	chr03:4,825,697..4829533	LOC_Os03g09250
*Myo-inositol 3-phosphate synthase-2*	*INO2*	Os10g0369900	chr10:11,624,392..11629513	LOC_Os10g22450
*Myo-inositol monophosphatase-1*	*IMP-1*	Os03g0587000	chr03:21,681,989..21685007	LOC_Os03g39000
*Myo-inositol monophosphatase-2*	*IMP-2*	Os02g0169900	chr02:3,792,694..3796762	LOC_Os02g07350
*Myo-inositol kinase*	*MIK*	Os07g0507300	chr07:19,258,741..19268283	LOC_Os07g32400
*Inositol 1,3,4,5,6-pentakisphosphate 2-kinase 1*	*IPK1*	Os04g0661200	chr04:33,735,145..33739378	LOC_Os04g56580
*Inositol 1,3,4,5,6-pentakisphosphate 2-kinase 2*	*IPK2*	Os02g0523800	chr02:19,121,903..19125625	LOC_Os02g32370
*Multidrug resistance-associated protein 13*	*MRP13*	Os03g0142800	chr03:2,367,856..2374437	LOC_Os03g04920
*2-Phosphoglycerate kinase*	*2-PGK*	Os02g0819400	chr02:35,170,411..35175254	LOC_Os02g57400
*Inositol 1,3,4-trisphosphate 5/6-kinase 1*	*ITPK1*	Os10g0103800	chr10:301,799..308024	LOC_Os10g01480
*Inositol 1, 3, 4-trisphosphate 5/6-kinase 2*	*ITPK2*	Os03g0230500	chr03:6,902,118..6907409	LOC_Os03g12840
*Inositol 1,3,4-trisphosphate 5/6-kinase 3*	*ITPK3*	Os03g0726200	chr03:29,535,973..29543273	LOC_Os03g51610
*Inositol 1,3,4-trisphosphate 5/6-kinase 4*	*ITPK4*	Os02g0466400	chr02:15,697,843..15699151	LOC_Os02g26720
*inositol 1,3,4-trisphosphate 5/6-kinase 5*	*ITPK5*	Os10g0576100	chr10:22,943,712..22945124	LOC_Os10g42550
*Inositol 1,3,4-trisphosphate 5/6-kinase 6*	*ITPK6*	Os09g0518700	chr09:20,243,654..20248528	LOC_Os09g34300

### Strategies to Develop Low-PA Grains with High Micronutrient Availability

PA inhibits the absorption of micronutrients, therefore reducing the PA content is important for improving the micronutrient bioavailability of cereals. A reduced PA content is effective in increasing the absorption of Zn (Egli et al. 2004) and Fe (Hurrell et al. 2003). Several physical, biological and biotechnological methods for reducing the PA content and increasing the bioavailability of essential nutrients have been reported (Fretzdorff and Brummer 1992; Shi et al. 2007; Ertas and Turker 2014).

PA can be eliminated from food by soaking and sprouting seeds, thereby activating endogenous phytase enzymes that hydrolyze PA (Lestienne et al. 2005; Kumari et al. 2014; Mahesh et al. 2015). Although soaking for 24 h at 30 °C could be used to increase Zn bioavailability, this method is not effective for Fe as such a treatment tends to leach Fe ions to the medium (Lestienne et al. 2005). Soaking followed by cooking is an effective means to significantly reduce the PA content of legumes (Huma et al. 2008). Processing techniques, for example milling, will eliminate PA but also remove the majority of minerals (Liang et al. 2008) and, thus, are not considered a suitable option for PA removal. Transgenic cereals that express and accumulate microbial phytase in seeds have been developed but these materials require laborious processing steps before the product can be fed to animals (Brinch-Pedersen et al. 2003). Therefore, more simple, economical and sustainable solutions are required (Kuwano et al. 2009).

Identifying *lpa* mutants impaired in PA biosynthesis or transport is another strategy for increasing the bioavailability of essential micronutrients such as Fe and Zn. Such mutants could significantly improve human nutrition and reduce environmental P pollution (Ockenden et al. 2004; Andaya and Tai 2005; Bhati et al. 2014). In this regard, understanding the functional and biochemical characteristics of the genes and gene products involved in PA biosynthesis in developing grains is essential. Next, we discuss possible strategies, the use of *lpa* mutants, transgenic technology and genetic markers and describe two key approaches: manipulating grain PA and micronutrient distribution and the PA/micronutrient ratio.

#### lpa Mutants

A possible approach for developing low-PA crops is to identify mutants (Kim et al. 2008). Mutations that block the synthesis or accumulation of PA during seed development are called *lpa* mutations and have been isolated from some important crops, including maize (Raboy et al. 2000; Pilu et al. 2003), barley (Dorsch et al. 2003), rice (Liu et al. 2007), soybean (Hitz et al. 2002), and wheat (Guttieri et al. 2004). The PA content of these mutants was reduced by 45–95% compared to wild-type seeds (Larson et al. 2000; Frank et al. 2009). In order to develop *lpa* plants, altering or inhibiting the first step of PA biosynthesis is proposed to be the most useful approach (Yuan et al. 2007; Kuwano et al. 2009). In rice, *lpa* mutant lines have been developed with significantly reduced levels of PA and increased Pi contents (Bryant et al. 2005; Kim et al. 2008). Comparable increases in Pi will be essential for maintaining panicle photosynthesis as a large amount of Pi is invested in the photosynthetic process.

The first *lpa* mutant rice, ‘Kaybonnet’ *lpa1–1*, a non-lethal single recessive mutant, was found to have an approximately 45% reduction in bran PA and a molar equivalent increase in Pi compared to wild type (Larson et al. 2000). Thereafter, several rice *lpa* mutants with 34% to 75% reductions in seed PA content were developed and characterized (Liu et al. 2007; Kim et al. 2008). The rice *lpa1* locus was fine mapped and further delimited to a 47-kb region between markers RM3542 and RM482 (Andaya and Tai 2005). Total P, Ca, manganese (Mn), and PA-P contents in whole grains were lower and the Zn content was higher in *lpa1* seeds compared with the wild-type, whereas the K, Fe and Mg levels were similar (Liu et al. 2004). Another *lpa* mutant of the *2-PGK* gene (*Os-lpa-XQZ-1*) developed from an *indica*-type rice, had significantly higher Fe (+16%), Zn (+19%) and Ca (+20%) levels and 12% – 35% reduction in PA than the wild type from all the locations tested (Frank et al. 2009). Os-*lpa*-XS110–1, a mutant of the *MIK* gene, had a 46% reduction in PA and increased levels of *myo*-inositol, raffinose, galactose and galactinol with no accumulation of lower inositol phosphates (Frank et al. 2007). Os-*lpa*-XS110–2, which is closely related to *OsMRP13,* a transporter of PA (Nagy et al. 2009), and is similar to an *lpa1*-type mutation in maize that is a multidrug resistance protein gene (*ZmMRP4*) mutant, observed with 23% reduction in PA and higher Pi content but does not accumulate lower inositol phosphates (Goodman et al. 2004; Frank et al. 2007; Liu et al. 2007). These mutants also show a variation in P fractions and micronutrients (Table 2). Such a combination of traits is desirable for enhancing mineral bioavailability, since chelation of minerals by PA would be reduced when the seed PA content is low.

**Table 2:** PA-P, Pi, Total P (TP) and Micronutrient Contents in some Mutants, Transformants and Wild Type Rice

Mutant/wild type/Transformant	PA-P (mg/g)	Pi (mg/g)	TP (mg/g)	Ca (mg/kg)	Fe (mg/kg)	Zn (mg/kg)	Reference
Mutant							
Os-lpa-XS-110-1	0.66–0.76	1.17–1.45	2.61–3.03	129–154	13.0–18.3	17.3–28.1	Frank et al. 2009 Liu et al. 2007
Os-lpa-XS-110-2	1.28–1.30	0.66–0.84	2.75–3.23	133–172	11.4–22.7	23.3–35.8	
XS 110 (WT)	1.82–2.08	0.17–0.23	2.8–3.34	125–161	13.2–17.8	19.3–31.9	
Os-lpa-XQZ-1	1.22–2.28	1.24–1.55	3.32–3.68	125–180	12.4–19.3	20.2–32.3	
XQZ (WT)	2.11–2.28	0.21–0.25	3.3–3.33	105–130	10.5–16.9	17.1–29.8	
Kaybonnet *lpa1–1*	1.28–1.45	0.86–0.97	3.24–3.62	101–115	13–16	22.0–25.0	Bryant et al. 2005
Transformant							
T4 IO6–97-4 & IO6–10-5	3.16–5.23	1.8–2.3	3.91–3.97	7.52^a^	12.61^a^	26.62^a^	Ali et al. 2013a
T3 196–11-6	4.273	2	3.939	7.196 ^a^	11.62^a^	24.13^a^	Ali et al. 2013b

WT-Wild type rice, ^a^indicates the values from milled seeds

With any newly developed trait, breeders are most concerned about the impact on final yield. In general, reduced germination, poor agronomic performance and yield reduction issues have been observed in mutant rice lines. (Raboy et al. 2000; Zhao et al. 2008). This low performance may have negatively affected the practical use of these lines in breeding programs. Both seed yield and seedling vigor of rice *lpa* mutants appear to be inferior to those of wild-type. In a study with *lpa* mutants compared with their wild-type parental varieties, a yield reduction of 12.5–25.6% and a 7.8–26.3% reduction in seed viability were reported (Zhao et al. 2008). These results highlight the inability to use these lines for breeding comparatively high yielding *lpa* varieties. In contrast, two *lpa* mutants, barley *lpa1–1* and soybean *Gm-lpa-ZC-2*, have not shown any yield reduction (Bregitzer and Raboy 2006; Yuan et al. 2007). Though the *lpa* rice lines developed through mutation have negative impacts on plant performance, studies on barley and soybean emphasize the possibility of developing suitable rice *lpa* mutants when more attention is given simultaneously to yield, yield-related traits and agronomic performance.

#### Transgenic Approaches

As conventional breeding attempts using mutants most often results in poor agronomic performance and low seed germination, the use of transgenic plants is a complementary approach to overcome these issues (Kuwano et al. 2006). In transgenic rice, reducing the PA content in seeds by suppressing *RINO1* gene expression driven by the *RINO1* or *CaMV35S* promoters resulted in an increase in available Pi but no reduction in the total seed P levels (Feng and Yoshida 2004). These investigators observed that the *RINO1* and *CaMV35S* promoters were also active in vegetative tissues in addition to developing seeds; however, the effects of the transgenes were low compared to the effects of the *lpa* mutants. Thereafter, seed-specific promoters from the rice major storage proteins *GlutelinB-1* (*GluB-1*) (Kuwano et al. 2006) and 18-kDa *Oleosin 18* (*Ole 18*) were used to suppress *RINO1* gene expression in rice seeds (Kuwano et al. 2009). The *Ole 18* promoter drives expression specifically in the aleurone layer and embryo of the seeds (Qu and Takaiwa 2004). The resulting transgenic lines showed strong *lpa* phenotypes with a 68% reduction in PA content, a concomitant increase in Pi and no negative effects on seed weight, germination or plant growth (Kuwano et al. 2009). However, silencing the *MIPS* gene might lead to detrimental alterations in important metabolic pathways utilizing *myo*-inositol, a compound that plays key roles in different plant metabolic pathways (Ali et al. 2013b). Therefore, to reduce the PA content in seeds, transgenic rice plants targeting a later stage in PA biosynthesis were generated by silencing the *IPK1* gene using the *Ole 18* promoter in an RNAi-mediated approach. The resulting transgenic rice plants had a substantial reduction in seed PA levels without hampering seed development or germination (Ali et al. 2013a). Accordingly, transgenic approaches offer several possible ways to develop rice lines with decreased PA contents (Table 2).

#### Use of DNA Marker Technology

Genetic DNA marker technology enhances the efficiency of plant breeding through marker-assisted selection of improved traits. Using these molecular techniques in future rice breeding should be an effective approach for reducing micronutrient deficiency issues; however, limited molecular level research studies have been carried out, and only a few Quantitative Trait Loci (QTLs) for PA content are known in rice. Two QTLs for PA content were identified from an ‘IR64’ X ‘Azucena’ mapping population that were localized to chromosomes 5 and 12 and explained 24% and 15% of the total phenotypic variation, respectively (Stangoulis et al. 2006). These results indicate clearly that grain PA concentrations are under genetic control. The QTL on chromosome 5 was common for PA and total P and was flanked by markers RM 305 and RM 178, whereas the other QTL for PA on chromosome 12 was flanked by RM 247 and RM 179. No genes related to PA biosynthesis were found on chromosome 5 nor on chromosome 12 (Table 1). There might be unknown regulatory factors regulating PA content in these chromosomal regions. The QTLs for PA content did not overlap with QTLs for Fe, Zn or Mn accumulation (Stangoulis et al. 2006), suggesting that using molecular markers in breeding could be used to modify the PA level without affecting grain micronutrient content. To date, there are no reports of using these markers in rice breeding programs.

#### Manipulation of PA and Micronutrient Distribution Patterns in Grain

The increasing global population requires an increase in rice yield with high micronutrient concentrations and its bioavailability to meet the nutritional demand. Rice grain typically consists of the hull and the inner edible portion; the average weight distribution of brown rice includes 89–94% starchy endosperm, 1–2% pericarp, 4–6% seed coat and aleurone and 2–3% embryo (Juliano 1972). In rice seeds, approximately 70% of the total seed P is found in the form of PA with approximately 80% or more present in the aleurone and pericarp and less than 10% in the embryo (O’Dell et al. 1972; Iwai et al. 2012). Approximately 90% of all seed PA is located in the aleurone layer and the remainder is in the embryo of many seeds, except for maize where more than 90% is stored in the embryo (O’Dell et al. 1972). PA accumulates in protein bodies called globoids, and a large number of these structures are present in the aleurone layer with fewer located in the starchy endosperm cells (Yoshida et al. 1999). A study investigating the effect of the degree of milling (DOM) on three *indica* rice cultivars reported variations in the distribution of PA and minerals in bran and the endosperm fraction of rice grains (Wang et al. 2011) (Table 3).

**Table 3:** Distribution of PA, Zn and Fe in Bran and Core Endosperm of three *indica* Rice Cultivars (Wang et al. 2011)

Cultivar	Bran			Core endosperm		
	PA %	Zn%	Fe%	PA %	Zn%	Fe%
ZN 7	38	15.46	50.83	2.15	53.35	23.3
ZN 60	44.32	17.29	55.68	2.44	61.75	17.92
ZN 34	59.82	14.05	48.49	4.31	66.82	35.04

A significant amount of nutritionally important mineral elements accumulates in rice bran (embryo and aleurone layers), whereas a lower amount is found in the endosperm (Lamberts et al. 2007). As most of the PA is present in the aleurone layer and, due to its chelating ability, there is a high probability of PA binding to minerals inside the aleurone layer. According to synchrotron-based X-ray micro-fluorescence (μ-XRF) investigations, the relative concentrations of Zn, Fe and K are in the following order: bran > hull > whole grain > brown rice > polished rice (Lu et al. 2013). Zinc and Fe concentrations in the bran are three-times and seven-times higher than that of the hull and endosperm, respectively (Lu et al. 2013). Therefore, grain processing results in a significant reduction in the micronutrient content of rice (Liang et al. 2008) in which approximately 40–45% of the total Zn, two-thirds of the total Fe, and most of the total K, Ca and Mn are removed during polishing (Lu et al. 2013). As rice is mainly consumed after polishing, this significant removal of nutrients from rice bran affects human nutrition. Consequently, manipulating the micronutrient distribution, that is reducing the amount in bran and increasing the amount in endosperm, will be another approach for improving human micronutrient intake. Using μ-XRF analysis, Iwai et al. (2012) revealed the dynamic spatial distributions of micronutrients in developing rice seeds. The fine mapping method revealed that Zn is distributed from the aleurone layer to the inner endosperm with more than one-half of the total Zn present in the endosperm; Fe is localized in the aleurone layer. Considering the embryo separately, Zn is mainly found in the central parts of the embryo (the plumule and radicle), whereas Fe levels are higher in the scutellum and hull (Lu et al. 2013). The total amount of mineral elements in *lpa* seeds was identical to those of wild type, and the decreased PA content in seeds did not affect the translocation of mineral elements from vegetative organs into seeds but did affect mineral localization (Sakai et al. 2015). Zn and Cu were primarily localized in the narrow space around the aleurone layer, whereas P and K mainly diffused from the aleurone layer into the endosperm of the *lpa* seeds; however, the distribution pattern of Fe in *lpa* seeds was similar to that of the wild type. However, the optimal amount of the micronutrient present in different parts of rice grains, the mechanism responsible for differences in the distribution of micronutrients, and the resulting effects arising with the changes in micronutrient distribution should be broadly considered during low-PA development process.

#### Manipulation of the PA/micronutrient Ratio in Grain

**Fig. 4:**
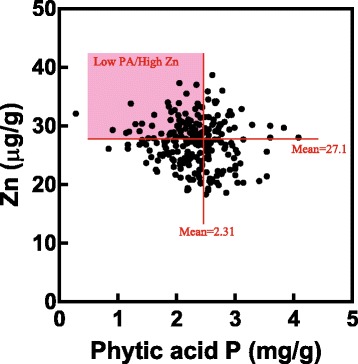
Relationship between PA-P and Zn concentrations in rice cultivars. The same data set as that used in Fig. 1 was used to construct this figure. Data for the nutrient concentrations of the edible portions of rice seeds of 18 rice cultivars were obtained without regard to their specific rachis position. Cultivars located in the red highlighted area are characterized by a low-PA content and a high-Zn content, two properties that are important for increased bioavailability

Effects of PA on mineral bioavailability also depend on the PA/micronutrient ratio (Frank et al. 2009). Zinc bioavailability is largely dependent on the PA/Zn molar ratios in humans (WHO 1996) and is inhibited when the molar ratio increases above 15:1 (Ma et al. 2007). Therefore, the PA/micronutrient molar ratio is a determinant for understanding mineral availability in food types (Frank et al. 2009). The molar ratio of PA/Fe is recommended to be 1 or lower for better Fe absorption from cereals when there are no enhancers for absorption (Hurrell and Egli 2010). In rice, grains in the primary rachis and upper rachises tend to have a lower molar ratio of PA/Zn relative to grains in the secondary rachis and lower rachises of the panicle, suggesting that grain position has an effect on Zn bioavailability (Su et al. 2014). To develop rice with a higher micronutrient bioavailability, it is wise to consider the PA/micronutrient ratio and set the target as a high micronutrient content as well as a low-PA content. The relationship between rice seed PA-P and Zn content from a published data set (Dietterich et al. 2015) is shown in Fig. 4. Both the PA and Zn contents varied mainly based on genotypic differences. Members of the group located in the quadrant corresponding to low-PA content and high-Zn content have higher Zn bioavailability; cultivars in the highlighted quadrant are expected to have the desired lower PA/Zn ratio. Searching for low-PA/high micronutrient cultivars from diverse rice germplasm could be a beneficial approach for finding a donor cultivar that can be used in genetic approaches to enhance grain micronutrient content as a strategy for improving the nutritional value of human diets.

## Conclusions

Sustainable solutions to improve mineral absorption and to overcome global micronutrient deficiencies will be mainly achieved through a combination of traditional and modern agricultural strategies. Plant breeding and molecular biological approaches are further required to reduce the effect of PA and increase the bioavailable micronutrient content of rice, while simultaneously promoting better agronomic and yield performances. Targeting the suppression of the PA biosynthetic genes, where the manipulation has no effect on physiological fitness, seems a fine approach for achieving low-PA crops. Further, focusing on manipulating the PA and micronutrient distributions in the endosperm and aleurone layer of rice grain, and the PA/micronutrient ratio appears to be a viable strategy to achieve rice biofortification.

Currently, information on the effect of elevated [CO_2_] on PA is limited but is needed to face the upcoming challenges of resulting nutrient deficiencies. Responsibility should be placed on developing breeding programs designed to alter the genetic makeup according to the challenges arising with elevated [CO_2_]. Identifying possible genotypes with a low-PA content and exploiting natural variation should be beneficial towards rice biofortification. Also, a better understanding of P absorption by roots and P translocation and accumulation in grains is vital for manipulating grain PA content. The effect of genetic (plant) and environmental factors and the impact from the manipulated genes to other traits should be broadly considered.

## Abbreviations

[CO_2_]: Carbon Dioxide Concentration
DAF: Days After Flowering
FACE: Free-air CO_2_ Enrichment
G6P: Glucose 6-phosphate
Ins(3)P1: 1D–*myo*-inositol 3-phosphate
InsP5: Inositol Pentaphosphate
InsP6: *myo*-inositol 1,2,3,4,5,6- hexa*kis*phosphate
lpa: Low-phytic Acid
PA: Phytic Acid
Pi: Inorganic Phosphate
QTL: Quantitative Trait Loci
RAP-DB: Rice Annotation Project Database
